# As to Drinking Water

**Published:** 1907-05

**Authors:** 


### As to Drinking Water.

WITH the present condition of the water supply in Buffalo, it is important to consider how to avoid infection. In other words, in the first place, how may the tap water be purified? and, in the second place, what is the purest and best water to substitute for the Niagara river water? The larger proportion of the population must, of necessity, drink from the hydrant water. For them the filter and teakettle are available, and properly used, afford as much protection as it is possible to obtain; but they must be used conjointly to bring complete success. If whenever it is impossible to boil and filter both, then all drinking water must be boiled. This dictate has been presented so often to the public through the newspapers and health department bulletins, that it would seem as if there were small need to repeat it here, but it also seems that we are prone to forget even the simplest rules of life unless we are reminded of them often, and sometimes by that saddest of all events,—death from preventable infectious disease.

Spring water has had a reputation for purity from an early day, but this traditional purity of the spring no longer is entitled to credit, because every well informed person knows that the source of the spring often becomes contaminated, and that it, also, is often insufficiently guarded from infectious germs, even in exceptional cases, perhaps, neglected altogether. So, if we not only would expect but insist upon absolute purity we must reject spring water, no matter how much its qualities in this respects are exploited nor how assertively advertised.

There is yet, however, left to us at least one source of water surely absolutely free from disease germs. We refer to water obtained by double distillation—a process which no pathogenic bacteria or in fact any other vegetable microorganisms can withstand or outlive. As an example of this water we may mention the products of the Hudor Company, of Buffalo, which is in fact the ideal drinking water of the present day. This company, it may be proper to mention, has introduced recently a new process of distillation whereby perfection as nearly as possible is obtained. The water as now prepared is "ozonized" as well as distilled, hence possesses an increased value for drinking purposes. This water is commended to physicians who can prescribe it with confidence; to the laity who are able to buy any water in the market it may be said to be the *sine qua non* of potable waters for domestic use.

The Apollinaris water, of course, cannot be surpassed as a carbonated water, and will continue to hold sway as the "queen of table waters." We have treated the subject of drinking water at this time only in a very general way, but expect to return to it in the early future to discuss it more in detail.

---

SOME manufacturers of foods and medicines have incurred the displeasure of the Secretary of Agriculture, by representing to the public by advertisements that their products are guaranteed as to purity by the government of the United States.

Secretary Wilson is reported to have said that "if this outrageous misrepresentation does not cease the department will publish a list bearing the names of manufacturers who are indulging in this campaign of deception."

The secretary in an interview declared that the serial number and guarantee required by the pure food and drug act to be placed on food and drug products were being used by these manufacturers for this purpose.

"The serial number," said Secretary Wilson, "is assigned to fix the responsibility where it belongs—upon the manufacturer—and to protect innocent dealers. It is the guarantee of the manufacturer, not the guarantee of the government."

The secretary declared that every effort would be made by the department to stop these statements.

"I will do a little advertising myself," he said, "in behalf of the people. I am growing tired of seeing these untruthful statements on the advertising pages of the magazines, the walls of the New York subway and the advertising space of the street cars of the principal cities.

"Manufacturers who will deceive the public about the guarantee will lie about the quality of their products."

He added that the law was to be administered fairly and that no honest manufacturer need fear that the department will take snap judgment on him or harass him in any way.

With the March issue of the Medical Mirror that journal as an independent publication ceased to exist, having been absorbed by the Medical Era of St. Louis. The Mirror will be best remembered as edited for a number of years by the late Dr. I. N. Love.

